# Incidence of severe rise in intraocular pressure after intravitreous injection of aflibercept with prefilled syringes

**DOI:** 10.1038/s41598-022-23039-6

**Published:** 2022-10-28

**Authors:** Vita Louisa Sophie Dingerkus, Gabor Mark Somfai, Stephan Kinzl, Selim Ismet Orgül, Matthias Dieter Becker, Florian Moritz Heussen

**Affiliations:** 1 Werner H. Spross Foundation for the Advancement of Research and Teaching in Ophthalmology, Zurich, Switzerland; 2 Department of Ophthalmology, City Hospital Zurich, Zurich, Switzerland; 3 Department of Ophthalmology, University of Heidelberg, Heidelberg, Germany; 4 Faculty of Medicine, University of Zurich, Zurich, Switzerland; 5 Department of Ophthalmology and Center for Clinical Research and Quality Assurance, Stadtspital Zürich, Birmensdorferstrasse 497, 8063 Zurich, Switzerland

**Keywords:** Macular degeneration, Ocular hypertension, Retinal diseases

## Abstract

Our aim was to analyze the intraocular pressure (IOP) changes following different intravitreous injection (IVI) procedures with or without prefilled syringes (PFS) and to elaborate their possible causes. Clinical study and laboratory assessment. 173 eyes of 141 patients. The IOP was prospectively measured pre- and postoperatively in three groups of patients receiving IVI either with ranibizumab (RP), aflibercept PFS (AP) or aflibercept vials (AV). The AP emptying volume (EV) was assessed using 40 aflibercept PFS vials: the plunger was aligned precisely (normal volume, NV) or right below the indication line (high volume, HV) and the drug was ejected with (wP) or without forced pressure (nP). Primary outcome was post-treatment IOP with type of IVI and pre-treatment IOP as fixed factors. Secondary outcome was identification of possibly confounding factors (age, sex, pathology, presence of pseudophakia, spherical error, and number of injections) and IOP > 30 mmHg post-treatment. An IOP rise above 30 mmHg was observed in 8/38 (22%), 16/51 (31%) and 35/86 (41%) cases in the RP, AV and AP groups, respectively (p = 0.129). Pre-treatment IOP was the only predictive variable for IOP rise (p < 0.001). The EV values in the NVnP, NVwP, HVnP and HVwP groups were 56.06 ± 10.32, 70.69 ± 4.56, 74.22 ± 7.41 and 81.63 ± 3.67 µl, respectively (p < 0.001). We observed a marked, although not significantly higher incidence of IOP elevations with the aflibercept PFS. One possible reason may be the error-proneness of administering the correct volume with the AP. Caution should be taken when using the aflibercept PFS in order to prevent potential optic nerve damage in cases with marked elevation in IOP.

## Introduction

The advent of intravitreous anti-vascular endothelial growth factor (VEGF) injection (IVI) therapies has revolutionized our understanding and treatment outcomes of macular diseases. Today, pre-filled syringes (PFSs) are available and have found wide-spread use in clinical practice. Ranibizumab and aflibercept have become available in Europe as PFS since their approval in March 2013 and April 2020, respectively.

IVIs with PFSs are supposed to reduce procedure time and improve patient safety due to a lower risk of endophthalmitis^1,2^. Postoperative rise in intraocular pressure (IOP) is suspected to lead to glaucomatous changes^3^ and there were informal reports that IOP can increase greatly following injections by the aflibercept PFS.

In June 2020, following the introduction of the new aflibercept PFS in our clinic, we observed an unusual incidence of severe spikes in intraocular pressure (IOP), leading to short-term transient visual loss in five eyes of five patients that were reported to Swissmedic, the Swiss agency for therapeutic products. Four of the five patients had diabetic retinopathy with macular edema, and one had wet age-related macular degeneration. All eyes had already been treated previously with intravitreous aflibercept vials without complications. Two of the five eyes had to undergo a paracentesis.

The exact emptying volume of PFSs has been assessed earlier describing notable differences^4–6^. Our hypothesis was that the drug itself, the design of the new PFS and/or the injection technique may result in inaccurate injection volumes.

Therefore, our aim was (1) to analyze the IOP changes following IVI procedures using PFSs and vials and (2) to assess the ejection volumes (EV) of the aflibercept PFS depending on the injection technique.

## Patients and methods

### Prospective observational study

We assessed the IOP data in consecutive patients receiving IVI either with ranibizumab PFS, aflibercept PFS or with an aflibercept vial (groups RP, AP and AV, respectively) between July 1 and August 28, 2020. The injections (all three types) were performed by 8 physicians who have been performing at least 100 injections per month. Most patients had already been treated by IVIs; however, 11 patients received their first injection (six eyes receiving RP, two AV, and three AP). All patients gave informed consent for the injection and data collection, the analyses were carried out in accordance with the tenets of the Declaration of Helsinki. Ethics approval has been obtained from the local ethics committee (Cantonal Ethics Board of Zurich; BASEC-No. 2020-02309). The injections were carried out according to the requirements of Swiss regulations at our medical retina outpatient clinic. During injections, the procedures set out by our standard operating procedures (SOP) and those on the labels of the injections' manufacturers (regarding the precise preparation of the PFS) were followed, however, a 33G MESORAM^®^ microneedle (C-M-T-S Dipl. Ing. Paul Wehrenpfennig, Munich, Germany) was used instead of the recommended 30G needle to administer the drug. Our routine pre-procedure protocol includes the administration of one drop of nepafenac (Nevanac^®^ 1 mg/ml), one drop of tetryzoline hydrochloride (Visine^®^), one drop of apraclonidine (Iopidine^®^ 1%) and three drops of oxybuprocaine (0.4%). The last drop to touch the eye before injection is the 10% povidone-iodine solution (or 0.04% polihexanide as an alternative).

The IOP was routinely measured pre- and postoperatively using a NIDEK® NT-530 noncontact tonometer. The postoperative IOP was measured within three minutes following the injection. In case of an IOP > 30 mmHg the measurement was repeated after 15 min. As a standard procedure at our clinic the patients were checked for hand motion vision immediately after the injection. Clinical characteristics of the patients were also recorded, including the ocular pathology, i.e. age-related macular degeneration (AMD), diabetic macular edema (DME), retinal vein occlusion (RVO), primary open angle glaucoma (POAG) and pseudoexfoliation syndrome (PEX) and other confounding parameters: age, sex, pseudophakia, hyperopia, myopia (in case of pseudophakia, only the preoperative refractive error was considered), number of injections and preoperative IOP. The postoperative IOP data in the RP, AP and AV groups were stratified as normal (below 30 mmHg) and elevated (above 30 mmHg). This cut-off was chosen in line with our primary care guidelines to indicate elevated pressure which may need close observation or short-term treatment.

### Assessment of the ejection volume

**Figure 1:**
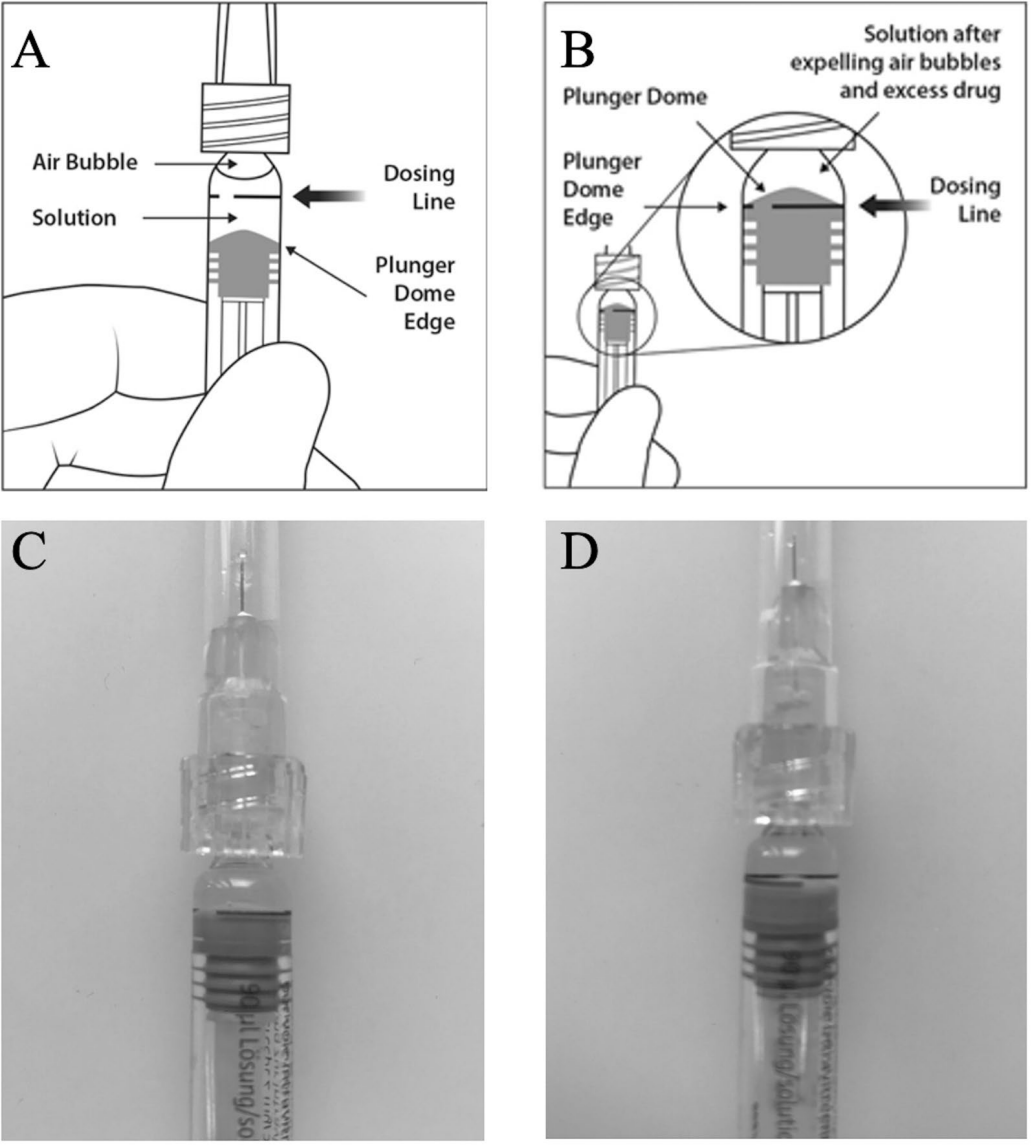
Injection technique with the aflibercept prefilled syringe (PFS). (**A**) Instructions for depressing the plunger rod to align the cylindrical base of the plunger dome edge with the black dosing line on the PFS. (**B**) and (**C**) Correct alignment of the plunger rod. (**D**) Incorrect alignment with the cylindrical base slightly beneath the dosing line. In a daily routine, perfect alignment may not always be achieved (Images **A** and **B** are taken from the manufacturer’s guideline: Eylea SmPC, © Bayer (Switzerland) AG).

In order to assess the exact fluid amount injected by the aflibercept PFS, the amount of the ejection volume (EV) was assessed using 40 original aflibercept PFSs provided by the manufacturer (Bayer AG, Zurich, Switzerland). We measured the EV in four different groups with 10 injections in each group: in the first two groups, the plunger rod was set precisely at the indication line (Normal Volume, NV) and the fluid was ejected without (nP) or with forced pressure (wP) at the end of emptying the syringe (NVnP and NVwP, respectively). In two further groups, the plunger was set right below the line with the plunger at tip (High Volume, HV) and was ejected without or with forced pressure (HVnP and HVwP, respectively) onto a Petri dish. (Fig. 1) Two experienced physicians (VLSD and GMS) performing more than 100 intravitreal aflibercept injections per month conducted the experiments with 5-5 injections in each group in a pharmacy-grade laboratory under controlled temperature and humidity conditions. A precision laboratory weighing scale (AX105 DeltaRange^®^, Mettler Toledo, Ohio) was used for the measurements of EV (calculated with a density of 1.034 mg/ml for aflibercept from the mass injected onto the Petri dish)^6^.

### Statistical analyses

Clinical characteristics between the three groups were tested with Kruskal–Wallis tests with Holm corrected Dunn’s post-hoc test for numerical variables and Fisher's exact test with Holm corrected pairwise Fisher’s exact tests for categorical variables. Pearson’s r was calculated to assess the correlation between preoperative and postoperative IOP. A one-way ANOVA was run to test for differences of pre- and post-treatment IOP between types of IVI. Because data for intraocular pressure (IOP) were partly clustered and thus, observations within patients were correlated, a random intercept model was run with type of IVI and pre-treatment IOP as fixed factors and post-treatment IOP as outcome. A second model was run including other possibly confounding factors (age, sex, pathology, presence of pseudophakia, spherical error, and number of injections). The assumptions of constant variance, normality, and agreement between fitted and observed values were checked with diagnostic plots. A second analysis was performed with a binary outcome, i.e. IOP > 30 mmHg post-treatment. A chi-square test and a mixed effects logistic regression were run to test the effect of type of IVI on IOP > 30 mmHg. A p-value of 0.05 was considered statistically significant.

The volume of the four different injection techniques were compared with a Welch’s ANOVA as variances were not equal. Normality was checked with a QQ-plot and Shapiro–Wilk normality test. Pairwise comparisons using t-tests with non-pooled standard deviation and Holm’s p-value adjustment was also performed. A ROC analysis was performed to assess the best cut-off of preoperative IOP for a postoperative IOP rise over 30 mmHg. Youden’s index with corresponding sensitivity and specificity was calculated.

All analyses were performed in the R programming language (version 3.6.2) (R Core Team, 2019). The package “nlme” was used to compute the linear mixed model, the package “lme4” was used to compute the mixed effects logistic regression, the package “tableone” was used to compute the descriptive statistics and to compare the groups with Wilcoxon rank sum tests or Fisher’s exact tests, and the package “ggplot2” was used to draw the figures. The package “pROC” was used for the ROC curve analysis.

## Results

### Comparing IOP changes with different anti-VEGF injections

We included 173 consecutively treated eyes of 141 patients in our study undergoing IVI at our retina outpatient clinic. Basic clinical characteristics of the three groups are shown in Table 1.

**Table 1:** Clinical characteristics with treated pathologies in the three patient groups injected with intravitreous anti-VEGF drugs.

	Aflibercept PFS	Aflibercept vial	Ranibizumab PFS	p value
n	86	51	36	
Age (median [IQR])	77.00 [66.00, 83.75]	76.00 [70.50, 81.00]	82.50 [79.25, 88.25]	0.001
Number of injections (median [IQR])	18.50 [8.25, 38.00]	21.00 [9.50, 35.00]	10.00 [2.00, 20.00]	0.016
DME (%)	20 (23.3)	14 (27.5)	4 (11.1)	0.177
RVO (%)	12 (14.0)	4 (7.8)	1 (2.8)	0.158
AMD (%)	52 (60.5)	32 (62.7)	30 (83.3)	0.040
POAG (%)	10 (11.6%)	3 (5.9)	1 (2.8)	0.263
PEX (%)	7 (8.1%)	2 (3.9)	6 (16.7)	0.124

The p value describes differences between the three groups in general. Only the median age was significantly higher in the ranibizumab PFS group than both aflibercept groups. All other charactreistics did not significantly differ within the three groups. *AMD* age-related macular degeneration, *DME* diabetic macular edema, *IQR* interquartile range, *POAG* primary open angle glaucoma, *PEX* pseudoexfoliation syndrome, *PFS* prefilled syringe, *RVO* retinal vascular occlusion.

Post-hoc pairwise comparisons indicated that age was significantly higher in the RP than in the AP group (p < 0.001). The number of injections was significantly higher in the AP vs AV and RP vs AP groups (p = 0.013 and p = 0.010, respectively). All other post-hoc comparisons were not significant.

An IOP rise to > 30 mmHg was observed in 40.7% in the AP group versus 31.4% in the AV, and 22.2% in the RP group, respectively. A simple chi-square test yielded a p-value = 0.129. A mixed effects logistic regression showed a similar result. Comparing a model with preoperative IOP and “with forced pressure” and “without forced pressure” group as fixed effects indicated that the model “with pressure” was not significantly better (p = 0.140). Comparing AV and RP to AP yielded p-values of 0.440 and 0.060, respectively.

**Figure 2:**
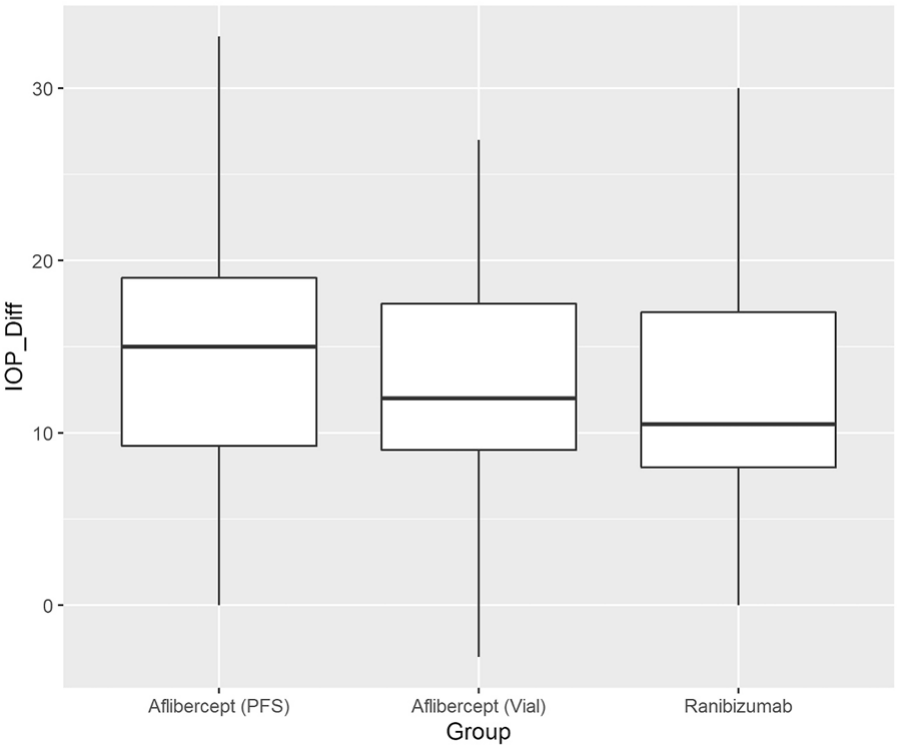
Boxplot of the intraocular pressure difference between the baseline (pre-injection) and the immediate (within 3 min) post-injection intraocular pressure by intravitreal injection type. The line in the box stands for the median, the lower and upper hinges correspond to the first and third quartiles. *IOP_Diff* Intraocular pressure differences, *PFS* prefilled syringes.

**Figure 3:**
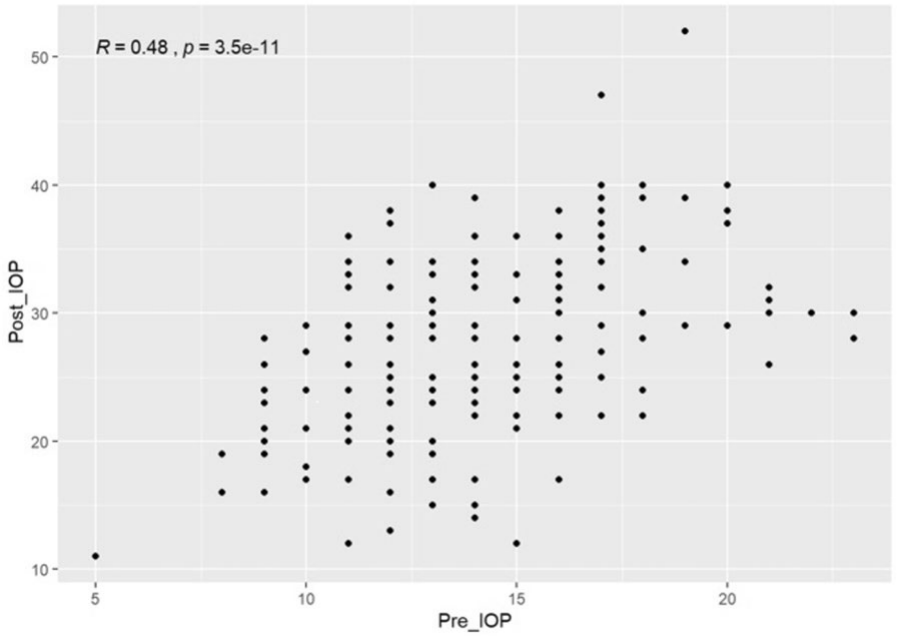
Scatterplot of intraocular pressure (IOP) pre- and post-treatment. Pearson correlation is shown in the upper left corner. *Post IOP* intraocular pressure after injection, *Pre IOP* intraocular pressure before injection.

**Figure 4:**
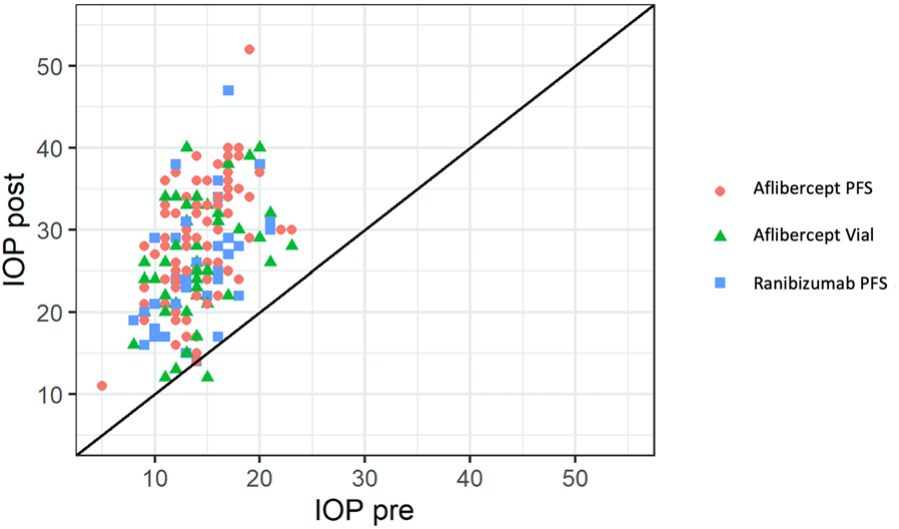
Scatterplot of intraocular pressures (IOP) pre- and post-treatment, stratified by type of intravitreous injection (IVI). Respective Pearson correlations were R = 0.55 (p = 0.001), R = 0.37 (p = 0.008) and R = 0.45 (p = 0.006) in the aflibercept PFS, aflibercept vial and ranibizumab PFS groups, respectively. Abbreviations: *IOP post* intraocular pressure after injection, *IOP pre* intraocular pressure before injection, *PFS* prefilled syringes.

Figure 2 shows the IOP differences measured postoperatively for three types of IVI. Mean IOP difference was 14.23, 12.59, and 11.89 mmHg for AP, AV, and RP, respectively. Table 2. includes the pre- and postoperative IOP values in the three groups. Figures 3 and 4 show the correlation between pre- and post-treatment IOP. In all cases with IOP increase the pressure normalized to almost preoperative values at the 15-min measurement by local conservative therapy; paracentesis was necessary in two cases.

**Table 2:** IOP characteristics pre- and postoperatively in the three patient groups.

	Aflibercept PFS	Aflibercept vial	Ranibizumab PFS	p value
n	86	51	36	
Pre_IOP (mean [SD])	14.21 (3.11)	13.82 (3.32)	14.06 (3.44)	0.798
Post_IOP (mean [SD])	28.44 (7.63)	26.29 (7.03)	25.94 (7.58)	0.129
IOP diff (mean [SD])	14.23 (6.45)	12.59 (6.59)	11.89 (6.78)	0.138

*IOP Diff* Intraocular pressure differences, *PFS* prefilled syringes, *SD* standard deviation.

The random intercept model indicated that pre-treatment IOP had a large effect on post-treatment IOP, but that type of IVI is not greatly affecting post-treatment IOP. This conclusion was not altered when including other possibly confounding factors (Tables 3 and 4).

**Table 3:** The random intercept model for the pre-treatment intraocular pressure (IOP).

	F-value	P-value
Intercept	2772.1230	< 0.0001
Pre-treatment IOP	51.2579	< 0.0001
Group	1.5633	0.2266

**Table 4:** Random intercept model with confounding factors.

	F-value	P-value
Intercept	1944.5147	< 0.0001
Pre-treatment IOP	49.4894	< 0.0001
Group	0.4675	0.6354
Age	0.7831	0.3783
Sex	1.7732	0.1860
No injections	2.3395	0.1460
Pseudophakia	0.0865	0.7728
Hyperopia	0.2926	0.5965
Myopia	0.0293	0.8664
DME	0.1014	0.7508
RVO	3.6737	0.0581
AMD	1.4528	0.2309
POAG	0.0513	0.8212
PEX	0.0212	0.8861
NA	0.4621	0.4982

*AMD* age-related macular degeneration, *DME* diabetic macular edema, *NA* narrow angles, *No Injections* Number of injections in the course of therapy, *POAG* primary open angle glaucoma, *PEX* pseudoexfoliation syndrome, *RVO* retinal vein occlusion.

**Figure 5:**
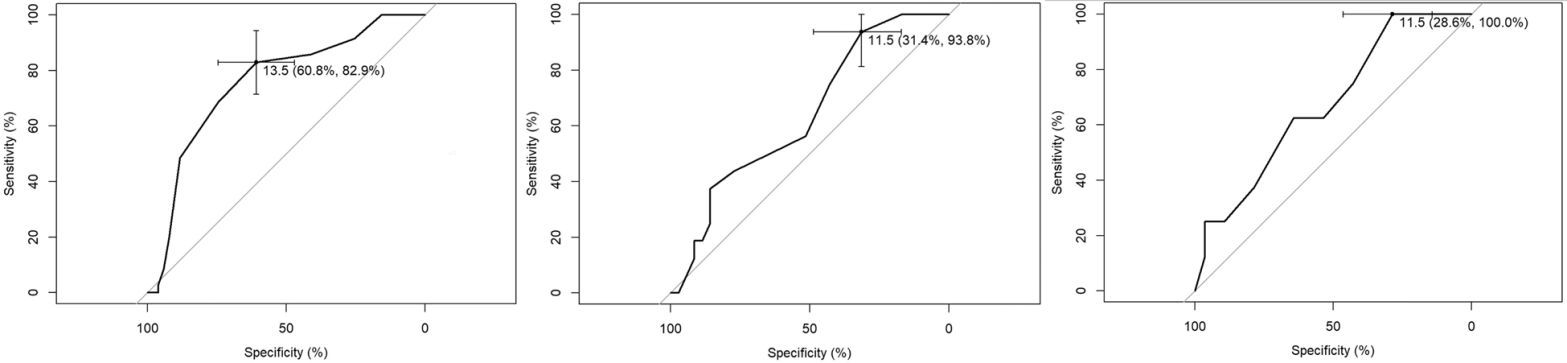
ROC curve and Youden’s index for pre-treatment intraocular pressure (IOP) and outcome increase in pressure > 30 mmHg, from left to right for the aflibercept prefilled syringes (PFS), aflibercept vials and ranibizumab PFSs.

The ROC curves for the IOP yielded cut-off values of 13.5, 11.5 and 11.5 mmHg for the EP, EV and RP groups with a sensitivity/specificity of 61/83%, 31/94% and 29/100%, respectively (Fig. 5).

### Comparing volumes of four injection techniques

**Figure 6:**
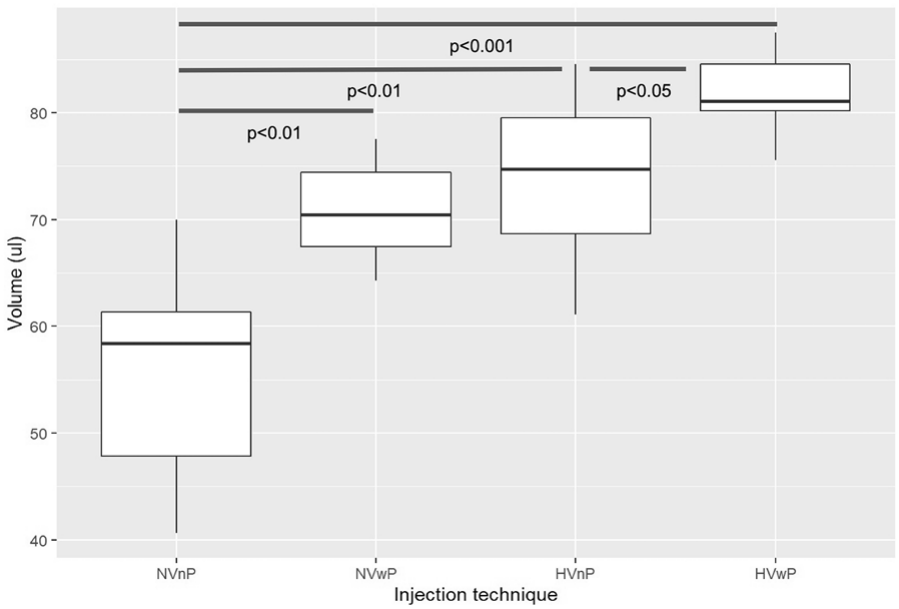
Boxplot of the distribution of volumes in the different groups of injection techniques. The line in the box stands for the median, the lower and upper hinges correspond to the first and third quartile. The four different groups are Normal Volume without forced Pressure (NVnP) or with forced Pressure (NVwP) at the end of emptying the syringe and High Volume without or with forced Pressure (HVnP and HVwP groups). The p-values under the blue lines indicate the pairwise comparisons using t-tests with non-pooled standard deviations (SD). Note that SD are higher within the groups in which no forced pressure at the end of emptying the syringe was used, indicating higher variability of ejection volumes.

Welch’s ANOVA showed that volumes were significantly different between the injection techniques (p < 0.001) (Fig. 6). Pairwise post-hoc tests indicated that only the volumes of NVwP and HVnP did not differ significantly from each other.

## Conclusion

Intravitreous injections are widely used in ophthalmology to deliver drugs acting directly on the retina. The numbers of IVIs are steadily increasing worldwide, in part due to demographic changes as well as due to better diagnostic tools available. However, safety is an important issue when considering these therapies, such as infectious endophthalmitis or postoperative IOP increase.

We previously observed a significantly higher incidence of moderate and severe IOP elevations following IVI with the aflibercept PFS, supported by verbal communication with other centers Switzerland-wide, which we could verify by our study. In the meantime, on March 12, 2021, the EMA issued a warning about the increased risk of intraocular pressure rise following Eylea^®^ PFS and concluded that “this may be caused by incorrect handling of the pre-filled Eylea syringes”^7^. The EMA reminded healthcare professionals using Eylea pre-filled syringes “how to correctly handle pre-filled syringes and the appropriate measures to mitigate this risk”^7^.

In a recent report, Karatsai et al. analyzed a series of 748 consecutive patients injected with aflibercept PFS and compared their IOP data with 565 patients who were treated with aflibercept from the vial^8^. They found an IOP elevation of 20 mm Hg occurring significantly more frequently in the PFS arm (n = 13) than in the vial arm (n = 3)^8^. They could not identify any reason for the high IOP elevation.

A markedly, although not significantly increased incidence of IOP elevations above 30 mmHg following IVI with the aflibercept PFS was observed in our study. The injection technique is a standardized procedure, and we could not identify any other confounding factor potentially leading to IOP elevations following intravitreal injections than preoperative IOP. Interestingly, there were markedly more RVO patients in the AP group (14%, 8% and 3% across the AP, AV and RP groups, respectively), with a higher number of previous injections in the AP and AV groups (a mean of 18.5 and 21 vs 10 injections in the AP, AV and RP groups, respectively). One may infer that the cumulative number of injections administered to and preoperative elevated IOP in a given eye may be linked to the risk for IOP-elevation. This is in line with previous findings concerning risk factors for sustained IOP elevations after anti-VEGF injections with suspected changes in outflow facility^9,10^.

A possible reason for our initial observation of increased pressure with Aflibercept PFS could have been the error-proneness of administering the correct volume with the AP. To assess this, we measured the ejection volumes of 40 aflibercept PFSs using four different settings, depending on the marker position and the ejection force used. We could indeed show that both the application of an incorrect plunger setting or a forced injection might lead to increased volumes, with the combination of the two reaching an almost doubling of the applied volume. This underlines the importance of the correct handling of the aflibercept PFS.

One reason for our observations could be higher diameter of the AP syringe, with a recent report by Hinkle and Hsu supporting this hypothesis^6^. They compared injection volumes relative to different plunger positions in ranibizumab and aflibercept PFS and concluded that small variations of the plunger setting have significant impacts on the injected volume, especially with aflibercept PFS^6^. Another recent study by Gallagher et al. assessed a lower volume expressed from a 1 ml syringe versus the aflibercept PFS^11^. In support of this, Pallikaris et al. demonstrated that there was a linear relationship between the injected volume and IOP rise^12^. In another recent report by Guest et al. the injection volumes by 12 physicians with the aflibercept PFS using incorrect plunger positions were 71% higher compared to the BD Luer-Lok 1-ml syringe, the latter showing significantly lower average volume deviation, as well^13^. Indeed, our results point toward the importance of the right injection technique with the aflibercept PFS to provide the correct amount of drug delivered intravitreously by making sure that the aflibercept PFS has its plunger properly aligned on the preprinted mark and no forceful pressure on the syringe is exerted.

It is noteworthy that after having observed a series of distinct pressure spikes with transient vision loss in five eyes that had been treated with aflibercept PFS at our department (which we reported to Swiss authorities), our injecting physicians were re-trained on the handling of the PFS correctly. This fact might be a reason for the absence of statistically relevant incidence of high postoperative pressures thereafter. Also, the occurrences might have led to a certain bias among the injecting ophthalmologists to prevent any further vision threatening IOP elevations after intravitreal injections.

A previous work by Loewenstein et al. has shown that the IVI volume delivered by using three different syringes varied considerably with 84% of the injections greater and 16% less than the intended 0.05 ml volume^4^. An earlier study by Moisseiev et al. claimed that the use of a low dead space plunger increased precision, while using a smaller size syringe (0.5 ml) resulted in higher accuracy compared to the widespread 1 ml tuberculin syringe when delivering a 0.05 mL dose^5^. Both studies suggested the use of PFS for anti-VEGF for achieving more accuracy.

However, it has to be pointed out that volumes of up to 100 µl, e.g. in the case of intravitreous high-dose aflibercept can be well tolerated without sustained IOP rise according to a study by You et al.^14^. Therefore, our results could not clearly explain our clinical observations. Yet, in the aforementioned study the mean number of injections was relatively low (7.9), which does not reflect the cumulative number of injections as a risk factor as we mentioned earlier. An ongoing clinical trial with 1016 participants investigating the safety of high dose (100 µl) aflibercept in patients with AMD might provide more reliable results (https://clinicaltrials.gov/ct2/show/NCT04423718). Also, while high dose injections can be well tolerated, itdoes cause at least a transient IOP rise.

Looking at the different designs of the PFS (aflibercept versus ranibizumab), especially their different diameters, we think that the ideal type of syringe might yet have to be developed.

The manufacturer recommends the use of a 30 Gauge needle for injection. In line with our standard operating procedures, we have used a 33 Gauge needle for all IVIs for years now, without any safety signals. According to literature, smaller needles are associated with less vitreous reflux and thus may lead to a higher postoperative IOP^15^. Still, as we used the same needle size for each type of anti-VEGF delivery, we do not believe that this might have had a great impact on our results.

Postoperative IOP rise is typically a brief effect with spontaneous normalization of IOP and is considered to be a common side effect of anti-VEGF IVI with a reported incidence of one third up to 89% of injections. However, about 11% of subjects seem to develop sustained IOP elevations which need ocular antihypertensive treatment^16–23^. In particular, the risk of sustained pressure rises seems to be higher in eyes at risk for glaucoma (with either ocular hypertension, predisposition to anterior ischemic neuropathy or RVO) and in case of numerous treatments^24–26^. Various pathomechanisms have been postulated to contribute to pressure rises after intravitreous anti-VEGF injections, such as mechanical blockage of the trabecular meshwork or inflammatory reactions^27–31^. A recent study using optical coherence tomography angiography has shown progressive capillary loss in both the optic nerve head and the macula over the anti-VEGF treatment course of 12 months, suggesting the role of IVI therapy in glaucoma progression^32^. The Youden’s index calculated from our data shows surprisingly low IOP values as a threshold for higher incidence of postoperative IOP rise > 30 mmHg (13.5 mmHG in the AP group versus 11.5 mmHg for both RP and AV groups). This points towards the potentially high importance of keeping the preoperative IOP under control, eventually by applying additional topical or even systemic therapy. Such a conclusion lies out of the scope of our study design and aim but would certainly merit further investigation. In fact, the guideline of the European Society of Retina Specialists suggest that the injecting ophthalmologist should always be aware of the risk of IOP spikes after every injection, although preoperative procedures to lower the pressure, be it manual or medical, remain controversial^33^.

Our study has certain limitations and strengths besides the ones already mentioned above. We did not plan to provide a comprehensive study for the possible causes of IOP elevation but wanted to investigate its potential roots. We used simple tools to assess our primary question which poses certain methodological limitations. Also, our patient pool is relatively low and inhomogeneous making it difficult to provide solid evidence. Still, we believe that we could reproduce our previous observation and could model the everyday use of the aflibercept PFS in our clinical routine. In contrast to studies based on retrospective data obtained from a central database (such as in the case of Karatsai et al.^8^) we gathered data prospectively. The proportion of patients represents a rough estimate of our practice which is the reason for the uneven proportion of subjects in the three groups. As at the time of our study there were no such data available, we were not aiming for a power analysis but were rather planning our study with a pilot nature. We used 30 mmHg as an arbitrary limit for severe IOP rise, similarly to our clinical practice at our casualty clinic which, we believe, depicts the real life settings of an injection clinic the best. One may furthermore consider, that according to the nature of this study, the injecting ophthalmologists at our clinic were not blinded when using different types of syringes. This fact, and additionally the fact that they knew about the previous cases of severe IOP rise after aflibercept PFS and were re-trained for its proper use earlier, may have led to a learning effect that explains why there were no further symptomatic pressure rises noted during our study, following the initial cases that we reported to Swissmedic. Non-contact tonometry is known to deliver imprecise measurements by elevated IOP that might have influenced our measurements; however, this influence is then evenly distributed fashion across the three groups. By the comparison of injection techniques, only two executors carried out the measurements which might have introduced some bias. However, we believe that with such a small number of injections carried by the original product it would have been impractical to include more physicians in the testing. Besides that, both executors were experienced, carrying out more than 100 injections monthly. Finally, the time elapsed for the IOP assessment following the IVI could vary to a certain extent as the measurements were carried out outside the injection room. This way patient mobility could have potentially influenced the time until the measurement but we believe this effect was minimal and evenly distributed along the study population.

To our knowledge this is the first study comparing acute IOP outcomes by the aflibercept PFS with both aflibercept vials or ranibizumab PFS, with results pointing toward a plausible connection between the use of aflibercept PFS and an increased incidence of IOP rise. The significant variance in the ejection volume of the aflibercept PFS depending on injection technique seems unlikely to be the reason for this IOP increase, while preoperative IOP was found to be significantly linked to IOP rise. This fact underlines the important role of maintaining optimal preoperative IOP values and justifies its routine measurement and any perioperative measures to lower it. According to the recommendation by Karatsai et al., it is advised to routinely control postoperative IOP in order to provide feedback and thus enable quality control of intravitreal injections^8^. This may lead to increased patient comfort and potentially less damage to the optic nerve from IOP spikes.

Further prospective studies with larger patient numbers are warranted in order to elucidate the exact reason behind our observations. Till then, in line with the recommendation by EMA, caution should be taken when using the aflibercept PFS to prevent IOP spikes and thus potential optic nerve damage in patients undergoing long-time anti-VEGF therapy.

## Data Availability

The dataset used during an/or analysed during the current study are available from the corresponding author on reasonable request.
